# The use of carotene-containing preparation in cows for the prevention of postpartum complications

**DOI:** 10.14202/vetworld.2021.1059-1066

**Published:** 2021-05-04

**Authors:** Tatyana Vladimirovna Zubova, Vladimir Alexandrovich Pleshkov, Oksana Vladimirovna Smolovskaya, Alexander Nikolaevich Mironov, Larisa Nikolaevna Korobeynikova

**Affiliations:** Zootechnical Faculty, Federal State Budgetary Educational Institution of Higher Professional Education “Kuzbass State Agricultural Academy”, Markovtseva Street, 5, Kemerovo, 650056, Russia

**Keywords:** carotene, diet, embryos, feeding, involution, uterine

## Abstract

**Background and Aim:**

It is known that metabolic disturbances in the animal body negatively affect parturition, uterine involution, and, ultimately, fertility, especially in cows, during the first insemination. Although the method of diet optimization with the use of a software package results in positive outcomes, adjustment is required for certain groups of animals (e.g., cows), in accordance with the period of the year. Every year, in the spring and autumn, blood is taken from animals to detect metabolic disorders, and then either the diet is balanced or, if the cows lack vitamins and minerals, they are administered to cows parenterally or with food. The aim of this study was to assess the efficiency of using carotene-containing preparation in the prevention of postpartum complications in cows.

**Materials and Methods:**

Before the start of the experiment, blood was collected from the animals of the experimental and control groups, with ten animals in each group, and its serum was examined for the presence of carotene. Low carotene content was found in the serum of all animals (from 0.2 to 0.25 mg%) with the norm within the range of 0.40-0.62 mg%. The cows of the experimental group were injected subcutaneously with the carotene-containing preparation 30, 20, and 10 days before the expected calving date at a dose of 10 mL per head. The carotene-containing preparation was a solution of crystalline β-carotene substance in deodorized sunflower oil. Moreover, the share of β-carotene was at least 0.18%. The drug was administered intramuscularly into the rump.

**Results:**

In the postpartum period, the retention of the placenta was observed in two animals of the control group. The uterine involution in the cows of the control group was 16.0 (p<0.05) days longer than that in the cows of the experimental group. The duration of placenta separation in the cows of the control group was on average 3.21 h longer (p<0.01) than that in the cows of the experimental group. The period from calving to the introduction of the embryo was 63.17±1.56 days in the control group and 48.3±0.83 days in the experimental group. The survival rate of embryos in the cows of the experimental group was 60%, and the period from the calving date to the introduction of the embryo averaged 48 days, which were 14.9 (p<0.05) days less than that in the cows of the control group.

**Conclusion:**

When the carotene-containing preparation was administered in a dose of 10 mL subcutaneously to cows 30, 20, and 10 days before the calving date, the blood carotene content increased, and the duration of the last stage and uterine involution decreased. The period from the calving date to the introduction of the embryo was reduced to 48.3±0.83 days, and the survival rate of embryos was 60%.

## Introduction

Comprehensive zootechnical and veterinary measures and timely detection and treatment of the obstetric–gynecologic diseases play an important role in improving the efficiency of using cattle breeding stock. Metabolic disorders and the shortage of macronutrients, trace elements, and vitamins in the diet of cows lead to reproductive system dysfunction. Pathological processes start developing in the genital organs and other organs of cows, which often results in animal sterility [1-4]. In formulating the diets for cattle, the nutritiousness of Vitamin A should be analyzed, since Vitamin A is required for normalizing the reproductive function in animals and for improving their resistance to diseases. However, the nutritiousness of fodder can only be normalized with the use of β-carotene [5-7]. It has been found that “balancing the diet in terms of the carotene content is often ineffective since carotene in the feed is an unstable compound [8].” Sokolova *et al*. [9] have observed “a positive effect of feeding β-carotene (the “GoKar” drug) on the biochemical blood parameters in highly productive cows.” The reasons for and the consequences of metabolic disorders in the organisms of dairy cows are also noted by other authors [10,11]. According to Kuzminova [12], “carotene, regardless of the Vitamin A level, strongly influences the reproduction processes.” Sizova [13] also noted that “the content of carotene and calcium in the blood is lower in the winter”. Kuzmich [14] also reported the positive effect of the Carolyn drug on the cows’ reproductive function. Carotene is the main precursor of Vitamin A, which is very important in the reproductive and immunological processes in animals [15]. The reproductive process leads to dynamic changes in metabolism and energy consumption, which may be the reason for the excessive production of free radicals (oxidants) formed during the physiological process of oxygen consumption [16]. The antioxidant effects of some nutraceuticals during the reproductive cycle with the high oxidative stress improve the reproductive function and reduce infections and other diseases of the genitourinary system [17]. In their studies, Johansson and Waller noted that the vitamin status in the blood and milk of the cows in organic dairy production could satisfy the needs for Vitamins A and E without adding synthetic vitamins, except for the calving period when the needs are high [18]. After introducing carrots into the diet of cows, Japanese scientists noted that “feeding carrots improved the IgG1 concentration in the plasma of the cows during parturition [19].” Therefore, more biochemical and hematological studies of cattle blood are needed [20-24]. The biochemical and hematological studies of cow blood are widely used for predicting or diagnosing antepartum and postpartum metabolic disorders in cows [25-28]. Most often, incomplete feeding in the dry period causes ovarian dysfunction [29,30].

The analysis of the blood serum biochemical studies at most farms of the Kemerovo region showed a deficiency of β-carotene and Vitamin A in the organisms of the animals in all seasons. At present, in the Russian market, drugs produced by foreign companies that contain carotenoids for broiler skin and egg yolk pigmentation are available; they contain up to 10% of synthetic carotene. The scientists of the Krasnoyarsk Research Veterinary Institute developed and tested a series of natural β-carotene-based injectable sterile drugs. In the methods for preventing postnatal complications, the use of harmless drugs that contain carotene is important. However, no literature data on the administration of carotene-containing preparations to cows are available in the Higher Attestation Commission (HAC) journals, Web of Science and Scopus databases. Therefore, the topic is considered to be relevant.

The aim of the present study was to assess the efficacy of the carotene-containing preparation in preventing postpartum complications in cows. The tasks of the study were as follows: Studying the physiological, biochemical, and hematological parameters of the cow blood at the beginning and end of the experiment; analyzing the diet of dry cows; determining the efficacy of the carotene-containing preparation in preventing postpartum complications in cows, and assessing the preventive effect of the drug on the embryo survival rate on the first administration after calving.

## Materials and Methods

### Ethical approval

Ethical control of scientific research was carried out in accordance with the Directive 2010/63/EU of the European Parliament and the Council of the European Union for the Protection of Animals Used for Scientific Purposes [31], as well as the Federal Law of the Russian Federation of November 21, 2011 No. 323- FL on the Basics of Protecting the Health of Citizens in the Russian Federation [32] for protecting the rights, safety and well-being of all subjects of research, ensuring the progress of the humanism atmosphere at the academy, improving methodological standards in research, and developing sensitive approaches to experimenting with animals. The research involved specialists with higher veterinary and biological education. For each of the experimental animals, an individual record was created in which all the manipulations during the experiment and up to its completion were reflected daily. When working with animals, experimenters followed safety measures.

### Study period and location

The experiment was performed from October 2018 to February 2019 at AE Mikhaylovskoe LLP in the Kemerovo region.

### Formation of animal groups

For the experiment, the authors had selected 20 cows in their dry period. These animals had been divided into two groups (experimental and control), with ten animals in each group. The animals had been selected using the method of pair analogs. In selecting the animals, their live weight, breed, calving age, milk yield, milk fat, physiological condition, and the date of expected calving had been considered (Table-1). For the two groups of animals, the same feeding and keeping conditions were organized; that is, they were kept in the usual conditions. During the experiment, the requirements for cows and the organization of their feeding were considered. The least valuable breeding animals were used; they had a strong constitution and stud fatness and were gynecologically healthy. Full-fledged feeding and balanced diets were maintained at the farm, and the fodder for the cows was not lower than class 1 (fodder, mixed fodder, mixed fodder raw materials, hay, haylage, and silage from forage plants) [33-35].

**Table-1:** Scheme of the formation of cows’ groups (n=10).

Index	Group	

	Experimental	Control
Breed	Black and motley	Black and motley
Live weight (kg)	505.8±9.4	508.5±9.08
Duration of productive use	3-4 lactations	3-4 lactations
Milk yield (kg)	5.413±98.8	5.387±91.2
Milk fat	4.1±0.4	4.0±0.3
Physiological condition	Satisfactory	Satisfactory
Date of expected calving	September 21, 2009-September 29, 2019	September 21, 2009-September 29, 2019

### Keeping conditions

The keeping conditions for the cows corresponded to the generally adopted zootechnical standards and the rules for cattle keeping for reproduction, rearing, and sale (the Order of the Ministry of Agriculture of the Russian Federation No. 551 dated December 13, 2016) [36]. The cows were kept in light and dry livestock buildings, where the necessary microclimate parameters were observed. Satisfactory sanitary conditions were maintained in the territory of the complex and the premises.

**Figure-1:**
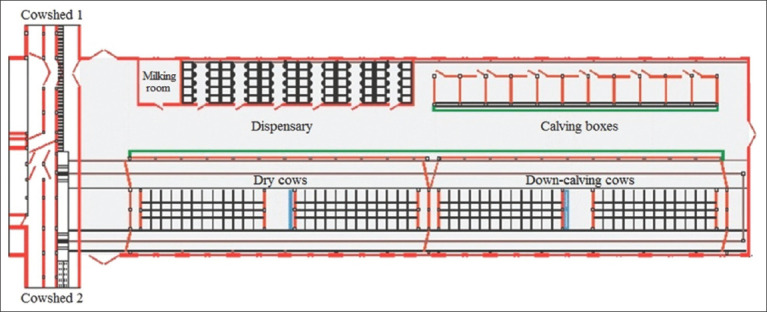
The maternity ward for keeping dry down-calving cows.

A maternity ward was used for keeping dry down-calving cows. The livestock capacity of the ward was 13-15% of the total number of animals. The maternity ward consisted of two separate compartments. Each compartment included the following sections: Prenatal section, parturition section, postpartum section, and a dispensary for calves (Figure-1). This technology ensured the veterinary welfare of the herd at the farm. To ensure the normal microclimatic conditions, in particular the air temperature (16°C), forced ventilation was installed for heating the incoming air.

Before being transferred to the prenatal section of the calving ward, the animals were sanitized after previously being fixed in special quarters. The cows were visually assessed, their live weight was determined, the skin was cleaned, and the back of their bodies was treated with disinfectants. The hoof horn was sanitized; formalin (3-5%) or copper sulfate (5-10%) disinfectants were used for this purpose. On the first signs of the upcoming calving, the external genitals of the cows were washed and disinfected with a 0.5% chlorhexidine solution, furacilin solution (1:5000), or potassium permanganate solution (1:1000). After treatment, the animals were transferred to the parturition section box.

In the maternity ward, the air temperature should be within the range of 14°C-18°C, the permissible air humidity 85% (optimal humidity level is 70%), the air velocity 0.2 m/s, and the microbial contamination not more than 50,000 m^3^. During the research, the air temperature in the maternity ward was 16°C, the air humidity 80%, the air velocity 0.2 m/s, and the microbial contamination 40,000 m^3^. The concentration of carbon dioxide was 0.12%, that of ammonia 8 mg/m^3^, and that of hydrogen sulfide 5.0 mg/m^3^. These indicators corresponded to the norm.

The carotene-containing preparation administration was started 30 days before the expected calving. After that, 20 and 10 days before calving, the drug was injected subcutaneously at a dosage of 10 mL per animal. The carotene-containing preparation (LLC Kompaniya Novie Tekhnologii, Kursk, Russia) is a solution of crystalline β-carotene substance in deodorized sunflower oil. With that, the share of β-carotene is at least 0.18%. The last injection was made in the maternity ward (10 days before calving) (Table-2). Before the experiment, blood was taken from the animals of the experimental and control groups, and its serum was analyzed for the presence of carotene. The drug was administered intramuscularly into the rump.

**Table-2:** Scheme of the experiment.

Group names	Animals	Preventive effect
Control	10	Without effect
Experimental	10	The carotene-containing preparation administration at a dosage of 10 mL per animal (30, 20, and 10 days before calving)

To exclude the symptoms of metabolic disorders in all animals, blood was obtained for biochemical and hematological studies. Quantitative indicators of erythrocytes (RBC), hemoglobin (HGB), mean corpuscular HGB concentration, mean corpuscular HGB, hematocrit, leukocytes (white blood cell), platelets, and leukogram were determined in the scientific research laboratory of “Biochemical, Molecular Genetic Research and Selection of Farm Animals” at the Kuzbass State Agricultural Academy in Kemerovo. The studies were conducted using an automatic hematology analyzer ABAXIS VetScan HM5 (USA) and ABAXIS reagents (USA).

The biochemical parameters of the blood of cows were determined on a semiautomatic biochemical analyzer MINDRAY BA-88A (MINDRAY Ltd., China). Before each blood sample was taken from the experimental animals, their physiological state was determined, and the body temperature, pulse rate, and number of respiratory movements per minute were measured (Table-3). The body temperature was measured with a mercury thermometer with the scale graded in Celsius from 34 to 42°C; the measurements continued for 5 min. The heart rate was measured on the external maxillary artery for 1 min. The respiratory rate was determined by the number of exhaling movements.

**Table-3:** The clinical and physiological parameters of the cows before the drug administration (n=10).

Value	Group	

	Experimental	Control
30 days before calving		
Body temperature, °C	38.5±0.29	37.94±0.31
Heart rate per minute	68.0±2.51	67.0±2.63
Respiration rate per minute	19.50±1.48	20.50±1.10
20 days before calving		
Body temperature, °C	38.24±0.30	38.54±0.37
Heart rate per minute	66.0±2.33	66.5±2.36
Respiration rate per minute	21.30±1.20	20.80±1.12
10 days before calving		
Body temperature, °C	38.94±0.31	38.94±0.16
Heart rate per minute	68.5±2.36	69.10±2.49
Respiration rate per minute	22.80±0.78	23.30±0.74

### Statistical analysis

The results were processed by the methods of variation statistics. The significance of the differences was determined on the basis of the Student’s t-test and indicated in the tables by the following signs: *at p<0.05, **at p<0.01, and ***at p<0.001. Digital material was processed using Microsoft Excel 2010 application package (Microsoft, USA).

## Results

The body temperature of the cows in the experimental group ranged from 38.24°C±0.30°C to 38.94°C±0.31°C. These values corresponded to the physiological norm. The changes in the heart rate of the cows in the experimental and control groups were within the physiological norm during all observations. No pathological changes in the respiratory movements were observed, and the respiratory rate in all periods of the study ranged from 19.50±1.48 to 23.30±0.74 respiratory movements per minute; that is, it was within the physiological norm. Blood was taken from the jugular vein 30 and 10 days before calving. Table-4 shows the hematological parameters.

**Table-4:** The hematological parameters of cow blood.

Value	Experimental group		Control group	
	
	Before the experiment (n=10)	After the experiment (n=10)	Before the experiment (n=10)	After the experiment (n=10)
Leukocytes, (WBC), 10^9^/L	9.5±0.17	6.1±0.11*	9.65±0.12	10.1±0.21
Leukocyte formula, %				
Basophils	-	-	-	-
Eosinophils	4.0	4.0	3.0	4.0
Neutrophils	36.0	43.0	36.0	39.0
Lymphocytes	57.0	45.0*	54.0	52.0
Monocytes	3.0	6.0	5.0	5.0
Erythrocytes, (RBC), 10^12^/L	6.8±0.42	5.9±0.12	6.9±0.07	6.2±0.07
HGB, g/100 mL	11.32±0.23	9.9±0.30	10.8±0.20	11.0±0.07
PLT, × 10^9^/L	428±31.11	532±32.24	456±32.4	443±28.71
HCT, %	41.33±0.21	40.24±0.15	41.43±0.33	40.32±0.57
The content of HGB in erythrocytes, pg	14.51±0.79	13.19±0.81	14.42±0.87	13.6±0.77
The concentration of HGB in erythrocytes, g/L	54.01±1.16	55.31±1.13	56.41±1.16	55.64±1.21

The difference at the beginning and at the end of the experiment was veracious at p<0.05 (*); p<0.01 (**). WBC=White blood cell, RBC=Red blood cell, pg=Picogram, HGB=Hemoglobin, HCT=Hematocrit, PLT=Platelets

As shown in Table-4, no significant difference was observed in the hematological parameters in the cows of the experimental and control groups before the experiment. However, by the end of the experiment, in the experimental group, the quantitative indicators of leukocytes had significantly decreased from 9.5±0.17 to 6.1±0.11x10^9^/L, or by 35.8% (p<0.01), whereas in the control group, these indicators slightly increased by 4.7%. The analysis of the leukocyte formula revealed a significant decrease in the number of lymphocytes in the cows of the experimental group from 57.0% to 45.0%, or by 21.1% (p<0.05). This might be due to the improved immunological state of the animals of the experimental group. The indicators of erythrocytes and HGB were within the physiological norm in all groups at the beginning and end of the study.

At the beginning of the experiment, the changes in the level of non-specific resistance in the experimental group of animals were as follows: Blood serum bactericidal activity (BSBA) 26.3%±3.2% and blood serum lysozyme activity (BSLA) 14.1%±0.72%. Subsequently, by the 30^th^ day of the research, a noticeable increase from 26.3%±3.2% to 65.8%±3.88%, or by 150.2% (p<0.001), was observed in BSBA, whereas BSLA remained unchanged (14.8%±1.51%) (Table-5).

**Table-5:** The biochemical indicators of cow blood (n=10).

Value	Experimental group		Control group	
	
	At the beginning of the experiment	At the end of the experiment	At the beginning of the experiment	At the end of the experiment
Total protein, g/L	73.27±0.84	77.14±1.51	73.89±1.8	74.43±1.4
Glucose, mmol/L	2.52±0.38	2.12±0.40	2.21±0.34	2.44±0.33
Calcium, mmol/L	2.77±0.12	2.81±0.17	2.68±0.12	2.64±0.15
Phosphorus, mmol/L	1.49±0.06	1.56±0.08	1.9±0.13	1.40±0.15
Alkalinity reserve, % CO_2_	55.5±3.2	58.3±2.0	56.0±2.6	53.3±1.7
Carotene, mg%	0.20±0.01	0.50±0.01***	0.25±0.04	0.28±0.05
Albumins, %	36.52±1.24	37.43±1.12	37.12±1.18	36.88±1.05
α-globulins, %	13.11±0.71	11.98±0.96	12.46±1.12	13.21±0.86
β-globulins, %	14.60±0.77	14.33±0.69	14.85±0.89	13.87±0.62
γ-globulins, %	35.77±0.81	36.26±1.2	35.57±1.14	36.04±1.28
BSLA, %	14.1±0.72	14.8±1.51	14.2±1.1	13.82±1.4
BSBA, %	26.3±3.2	65.8±3.88***	28.1±2.6	29.3±1.21

The difference at the beginning and at the end of the experiment was veracious at p<0.001(***). BSLA=Blood serum lysozyme activity, BSBA=Blood serum bactericidal activity

The indicators of the changes in the non-specific resistance level in the cows of the experimental group should be related to the activation of the immune hormonal system. Thus, after the carotene-containing preparation was injected, the neuroendocrine system influenced the activity of the non-specific immunity factors, which, in turn, had an opposite effect on its performance. The content of carotene at the beginning of the experiment was 0.20±0.01 mg/%, and that at the end of the experiment was 0.50±0.01 mg%; that is, this indicator increased in the experimental group by 150.0% (p<0.001). Furthermore, no significant difference was observed for other indicators. The content of carotene on average amounted to 0.22 mg% a day after the first administration, 0.30 mg% a day after the second administration, and 0.50 mg% after the third administration (Table-6).

**Table-6:** The content of carotene in cows blood in the experimental group after the drug administration.

Indicator	The day of the drug administration (days before calving)		

	30 days	20 days	10 days (the maternity ward)

	mg%	mg%	mg%
M	0.22	0.30	0.50
σ	0.02	0.02	0.04
Cv, %	11.07	6.77	8.49
m	0.01	0.01	0.01

The arithmetic mean (M), the standard deviation (σ), the coefficient of variation (Cv), and the arithmetic mean error (m)

Successful gestation required organizing individual normalized feeding of the cows, given the changes in live weight and milk productivity and the nutritious requirements for the months of lactation. At the farm, the following fodders and fodder additives that were used for feeding the animals were available: Spring wheat straw, high-quality pasture, and meadow hay of the first mowing (mowed at the beginning of the earing phase); raw potatoes, haylage of vetch and rye, oats, and high-quality wheat (mowed at the beginning of flowering); fodder yeast (crude protein 34%); dried beet pulp; sunflower meal (crude protein 32%, crude fiber 21%); and rolled barley, rolled wheat, monocalcium phosphate, zinc oxide, common salt, and Vitamin D (Table-7).

**Table-7:** The diet of the dry cows, the planned milk yield of 6000 kg, the live weight of 600 kg.

Composition	In the diet	Amount in kg	Amount with losses in kg
Rolled wheat	1.800	1.80	1.82
Sunflower cake CP 32%, CF 21%	3.000	3.00	3.03
Fodder yeast, CP 34%	0.500	0.50	0.51
Common salt	0.100	0.10	0.10
Monocalcium phosphate	0.300	0.30	0.30
Haylage of vetch/R/O/W 2 MOW. HQ BF	16.130	16.10	16.26
Raw potatoes	9.290	9.30	9.39
Pasture/meadow hay 1 mow. 50N HQ BE	2.840	2.80	2.83

The amount of the diet: 33.96 kg. Feeding days: 1. The fodder needs: 33.96 kg

The diets were optimized using the Korm Optima Ekspert software complex (version 2019.15.1) developed by the Kormoresurs company (Voronezh). At the end of the studies, the clinical observation of the parturition and postnatal periods in the experimental and control groups was conducted. In the experiment, the following parameters were considered: The duration of the placental stage, the duration of uterine involution, the period from calving until the embryo introduction, and the embryo survival rate (Table-8). In the normal animal feeding and keeping conditions, the cow’s placenta separated in 6 h after delivery.

**Table-8:** The indicators of the postpartum period in the cows.

Value	Experimental group (n=10)	Control group (n=10)
The duration of the placental stage, hours	5.0±0.12**	8.21±0.33
The duration of uterine involution, days	32.1±0.61*	48.1±2.15
The period from calving until the embryo introduction, days	48.3±0.83*	63.17±1.56
The embryo survival rate, animals/%	6/60	4/40

The difference between the groups was veracious at p<0.05 (*): p<0.01 (**)

A variety of factors influenced placenta retention in cows: Genetic, feed, immunological, and pathological factors. However, the etiology of this pathology remained understudied.

In the control group, placenta separation was observed in two animals within 9 h. In the cows of the experimental group, the duration of placenta separation was 5.0±0.12 h, and that in the cows of the control group was 8.21±0.33 h. On average, the duration of placenta separation in the cows of the control group was 3.21 h longer (p<0.01) than that in the cows of the experimental group.

Overall, 3 weeks were needed to restore the uterus to its normal size characteristic of cows before the onset of pregnancy. Usually, this time varies from 40 to 50 days. On uterine involution, its contraction occurs along with caruncle rejection and endometrium regeneration. In turn, the duration of the involutional processes in the uterus depended on the parturition period and the duration of placenta separation. The uterus involution of the cows in the control group was 16.0 (p<0.05) days longer than that of the cows in the experimental group. In the control group, the embryo survival rate after the first insemination was 40%. Moreover, the period from calving until the embryo introduction on average was 63.17±1.56 days. The embryo survival rate was 60%, and the period from calving until the embryo introduction on average was 48 days, which was 14.9 (p<0.05) days less than that in the control group.

## Discussion

As discussed in this study, domestic and foreign scientists are engaged in the research of Vitamin A deficiency in the diets of cows. A number of highly effective fodder additives have been proposed, such as the protein-carbohydrate vitamin and mineral fodder additive Golden Felucen [37]. The additive contains calcium, cobalt, iodine, and Vitamins A, D_3_, and E. The additive may additionally contain manganese, magnesium, and selenium. Moreover, antagonistic relationships between individual elements that negatively affect the regulatory mechanisms of these elements were observed [37].

Scientists have tested and proposed a method of using a mineral and vitamin premix to stabilize the physiological state of cows [38]. The premix contains manganese, zinc, copper, phosphorus, vermiculite, and Vitamin A and is formulated for the different phases of lactation of dairy cows. The disadvantage of this fodder additive is the incorrect consideration of the physiological norms of the need for certain microelements (manganese or zinc). Foreign scientists have noted that the additional introduction of vitamin and mineral preparations into the diet or their parenteral administration also contributed to the normal course of the postpartum period, the prevention of disorders, and the increase in the level of animals’ fertilization [39,40].

The main reason for ovarian dysfunction is considered to be unfavorable changes in the metabolic status in cows, caused by negative energy balance in the early postpartum period or induced by inadequate feeding [29,30]. Dmitrieva proposed the use of the Carofertin drug for dry cows, which has a positive effect on parturition due to the normalization of the level of carotene and Vitamin A and the calcium-phosphorus ratio and indirectly indicates the normalization of hormonal regulation of the parturition. Carofertin stimulates the clinical signs of estrus and ovulation, reduces the insemination index, increases the level of fertility, and increases the immunity of newborn calves by increasing the concentration of β-carotene in the colostrum of newly calved cows. It should be noted that Carofertin is registered in Russia, as well as in 14 European countries; its effectiveness has been proven by many years of clinical trials all over the world [41].

## Conclusion

As a result of administering the carotene-containing preparation in a dose of 10 mL to the cows in the experimental group (30, 20, and 10 days before the calving date), the duration of the last stage was reduced by 3.21 h (p<0.01). Accordingly, the uterine involution in the experimental group ended 16.0 (p<0.05) days earlier than that in the control group did (32.1±0.61 and 48.1±2.15 days). Overall, the survival rate of embryos after the completion of the uterine involution in the cows of the control group was 40% and that in the cows of the experimental group 60%.

## Authors’ Contributions

TVZ, VAP, and OVS: Conception and design, acquisition of data, analysis, and interpretation of data, drafting the article, revising it critically for important intellectual content, final approval. ANM and LNK: Conception and design, acquisition of data, analysis, and interpretation of data, drafting the article. All authors read and approved the final manuscript.
